# Systematic review and meta-analysis of Mental Health First Aid training: Effects on knowledge, stigma, and helping behaviour

**DOI:** 10.1371/journal.pone.0197102

**Published:** 2018-05-31

**Authors:** Amy J. Morgan, Anna Ross, Nicola J. Reavley

**Affiliations:** Centre for Mental Health, Melbourne School of Population and Global Health, The University of Melbourne, Parkville, Victoria, Australia; Central Queensland University, AUSTRALIA

## Abstract

**Objective:**

To provide an up-to-date assessment of the effectiveness of the Mental Health First Aid (MHFA) training program on improving mental health knowledge, stigma and helping behaviour.

**Design:**

Systematic review and meta-analysis.

**Methods:**

A systematic search of electronic databases was conducted in October 2017 to identify randomised controlled trials or controlled trials of the MHFA program. Eligible trials were in adults, used any comparison condition, and assessed one or more of the following outcomes: mental health first aid knowledge; recognition of mental disorders; treatment knowledge; stigma and social distance; confidence in or intentions to provide mental health first aid; provision of mental health first aid; mental health of trainees or recipients of mental health first aid. Risk of bias was assessed and effect sizes (Cohen’s d) were pooled using a random effects model. Separate meta-analyses examined effects at post-training, up to 6 months post-training, and greater than 6 months post-training.

**Results:**

A total of 18 trials (5936 participants) were included. Overall, effects were generally small-to-moderate post-training and up to 6 months later, with effects up to 12-months later unclear. MHFA training led to improved mental health first aid knowledge (ds 0.31–0.72), recognition of mental disorders (ds 0.22–0.52) and beliefs about effective treatments (ds 0.19–0.45). There were also small reductions in stigma (ds 0.08–0.14). Improvements were also observed in confidence in helping a person with a mental health problem (ds 0.21–0.58) and intentions to provide first aid (ds 0.26–0.75). There were small improvements in the amount of help provided to a person with a mental health problem at follow-up (d = 0.23) but changes in the quality of behaviours offered were unclear.

**Conclusion:**

This review supports the effectiveness of MHFA training in improving mental health literacy and appropriate support for those with mental health problems up to 6 months after training.

**Trial registration:**

PROSPERO (CRD42017060596)

## Introduction

Up to 1 in 5 adults will develop a mental health problem in any year[1] and because of this high prevalence, members of the public are likely to have contact with someone who has a mental health problem. The Mental Health First Aid (MHFA) course was developed in 2000 to teach community members first-aid skills to support people with mental health problems [2]. Developed in partnership between Betty Kitchener, an educator and mental health consumer, and Professor Tony Jorm, a mental health researcher, it adapts a familiar model from physical first aid training for injuries and emergencies to mental health problems. Mental health first aid is the help provided to a person who is developing a mental health problem or who is in a mental health crisis, until appropriate professional help is received or the crisis resolves. The course teaches how to recognise the clusters of symptoms of different mental disorders and mental health crises, how to offer and provide initial help, and how to guide a person towards appropriate treatments and other supportive help. This is summarised via the ‘ALGEE’ Action Plan:[3] Approach the person, assess and assist with any crisis; Listen and communicate non-judgmentally; Give support and information; Encourage the person to get appropriate professional help; and Encourage other supports.

From its beginnings in Australia, the MHFA program has spread worldwide with more than 22 countries adopting the program, and over 2 million people attending a course [4]. The Standard adult course is 12 hours long and includes information on depression, anxiety problems, psychosis, substance use problems, and crisis situations (e.g. suicide and self-harm, panic attacks, drug/alcohol overdose). As well as the Standard MHFA course for adults, there is a tailored course for adults assisting adolescents (Youth MHFA) which contains additional teaching about eating disorders. There are also specialized courses for various cultural and professional groups, including Aboriginal and Torres Strait Islanders, tertiary students, financial counsellors and pharmacists. These courses all teach how to give mental health first aid using the ALGEE Action Plan and include appropriate adaptation towards cultural or workplace contexts. MHFA courses are typically delivered via face-to-face instruction, but online/CD-ROM versions have also been developed. MHFA Instructors undergo rigorous selection procedures, training, and annual accreditation. Underpinning the MHFA program is a strong commitment to delivering training based on research evidence [5]. All course content is as evidence-based as possible and undergoes regular revision to incorporate new knowledge. Course materials draw on expert consensus studies that systematically combine the views of consumers, carers and professionals on how to provide MHFA (e.g [6–8]). This commitment to research evidence is also reflected in the continued focus on course evaluation.

A meta-analysis of 15 studies evaluating MHFA found that the program improves mental health knowledge, reduces stigmatising attitudes, and increases helping behaviours [9]. However, this review only included 6 controlled trials, 3 of which were conducted by the developers of MHFA. It also didn’t evaluate several outcomes of interest, including confidence and intentions to provide MHFA, MHFA knowledge, and the quality of MHFA behaviours provided to a person with a mental health problem. Since this review was conducted, a considerable number of new trials have been published evaluating the program. A new meta-analysis is warranted in order to include these new studies, evaluate the full range of training outcomes, examine the persistence of effects, and to further explore whether effects vary according to study characteristics. Therefore, this review aimed to provide an updated evaluation of the effectiveness of the Mental Health First Aid training program on improving mental health knowledge, stigmatising attitudes and helping behaviour.

## Materials and methods

This review was conducted in accordance with the PRISMA recommendations for systematic reviews[10] and the review protocol was registered with PROSPERO (CRD42017060596).

### Study eligibility criteria

Studies were eligible if they were randomised controlled trials (including cluster-randomised), quasi-randomised controlled trials, or controlled trials, in adults aged 18+. We excluded uncontrolled trials given their greater risk of yielding a biased estimate of effect [11]. We included studies evaluating the MHFA program if it was the primary intervention and not a component of a larger intervention. Both the standard adult course and its variants (e.g. translations or adaptations for specific populations) and the youth mental health first aid course were included. All modes of delivery were eligible for inclusion, including face-to-face, online/CD-ROM, and blended. If two versions of MHFA were evaluated within the one study (e.g. face-to-face or online), we selected the program that was more frequently evaluated in the included studies to avoid double-counting control group participants [11]. Studies could include any comparison condition, including waitlist, no intervention, a generic health education intervention, or other mental health education intervention. If there were multiple comparison conditions, we selected the comparison that most closely matched the intervention condition (e.g. in length or dose). We included studies that assessed the impact of MHFA on at least one outcome including knowledge, attitudes, perceived confidence or intentions to provide help, mental health first aid behaviours, and mental health symptoms. We planned to include studies in any language if they could be adequately translated into English using Google Translate. We identified only one such study [12], but as this was also reported in English in a separate publication [13], we used the English version. Unpublished or ongoing studies were eligible if the authors were able to provide us with outcome data.

### Identification and selection of studies

**Fig 1:**
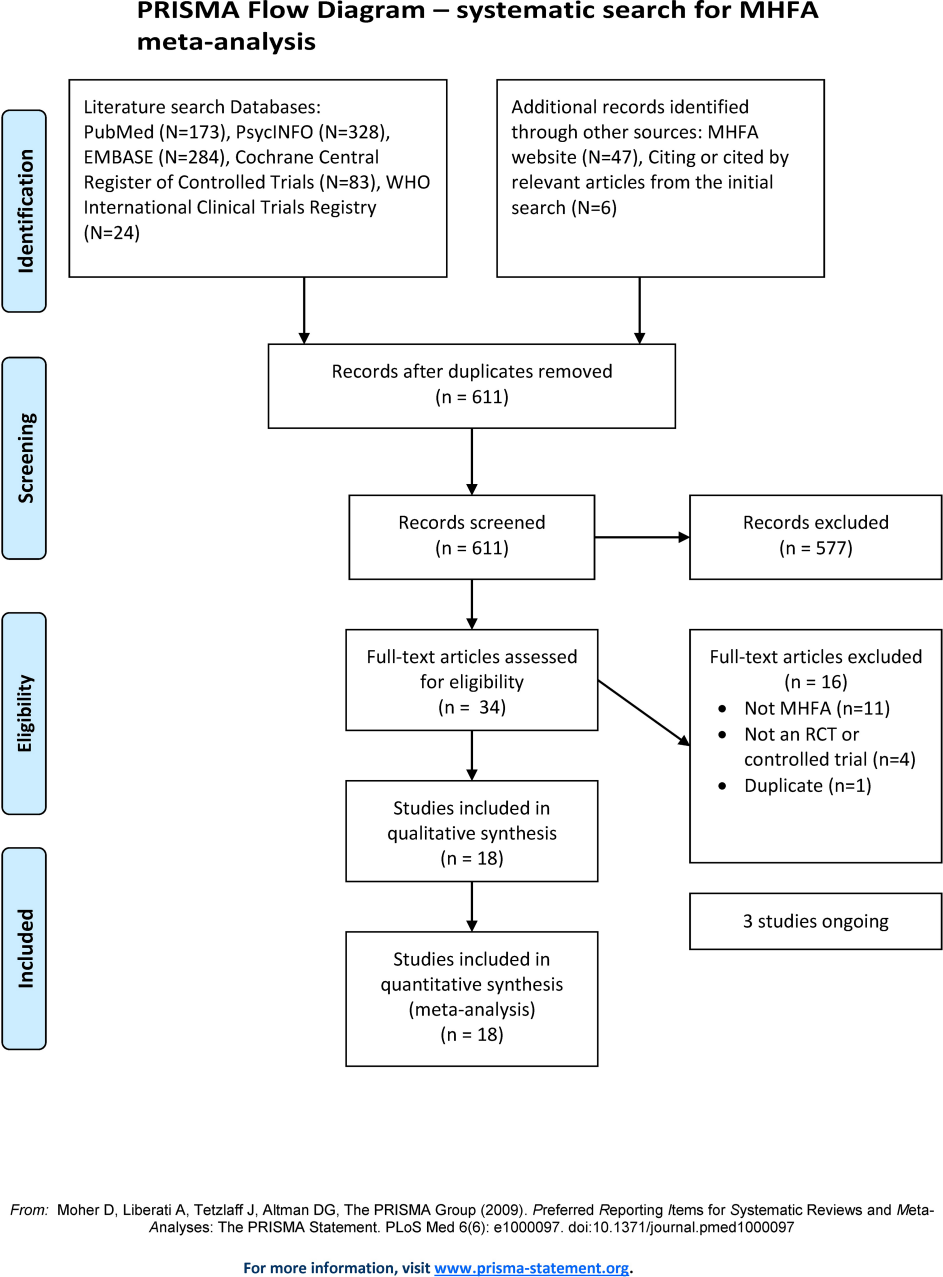
PRISMA flow chart.

A systematic search of the literature was conducted for studies published between the year of MHFA inception (2000) and 5^th^ April 2017. This systematic search was also re-run on 12^th^ Oct 2017, to detect any additional studies published since the initial search. We searched PubMed, PsycINFO (OVID interface), EMBASE (OVID interface), and the Cochrane Central Register of Controlled Trials (Wiley interface) for eligible studies. Specific search strategies were developed for each database, using a combination of both key words and MeSH/Map terms to include the following: ‘controlled trial’, ‘mental health’, ‘mental disorder’, ‘first aid’, ‘mental health training’, ‘MHFA’, ‘mental health first aid’, limiting to studies involving humans (see supplementary S3 Table for search terms used in each database). These database searches were supplemented by searching for trial protocols through the International Clinical Trials Registry Platform Search Protocol (http://apps.who.int/trialsearch/) and by examining the list of MHFA course evaluations provided on the MHFA International website (https://mhfa.com.au/research/mhfa-course-evaluations). The reference lists of included studies and relevant reviews identified through the search were also checked. One author (AR) screened titles and abstracts for potential inclusion and two authors (AR, AJM) independently screened the full-text of retrieved articles, with discrepancies resolved by consensus or arbitration from a third author (NJR). The flow of studies is presented in Fig 1.

### Data extraction and assessment of risk of bias

A data extraction template was developed and piloted. Study characteristics and outcome data from each study were extracted independently by two authors, with disagreements resolved by discussion. We attempted to contact authors to resolve any uncertainties or to obtain missing information, and received information from 10 studies [13–22]. Information extracted from each study included participant characteristics (% female, age [mean, SD, range], education level); intervention characteristics (name of program, length of program in hours, number of sessions, delivery mode [online, face-to-face, blended]; and study characteristics (design [controlled, RCT, cluster-RCT], comparison condition [waitlist, no intervention, generic health education intervention, other mental health education intervention], study population [university student, employee, member of the public], study size [total, intervention/control], follow-up occasions, country, publication status [unpublished data, peer-reviewed, not peer-reviewed], involvement of MHFA founder [yes/no]).

Four sets of primary outcomes were evaluated: (1) knowledge, (2) stigmatising attitudes, (3) confidence and intentions in providing MHFA, (4) provision of mental health first aid. These outcomes are not measured as part of MHFA training but have been evaluated in research trials as they measure the main constructs of the training.

#### Knowledge

Three kinds of knowledge outcomes were extracted, where available. Mental health first aid knowledge, when measured objectively, typically by true or false questions based on course content (e.g. *It is not a good idea to ask someone if they are feeling suicidal in case you put the idea in their head* and *It is best not to try to reason with a person having delusions*). These questions have face validity but no other psychometric properties have been reported. Recognition of mental health problems was based on accuracy of identification of mental health problems described in a short vignette, most commonly depression or schizophrenia. These vignettes were written to satisfy diagnostic criteria and correct recognition has been validated by an Australian national sample of clinical psychologists, psychiatrists, and general practitioners (GPs) [23]. Beliefs about effective treatments for mental health problems was assessed by the extent to which participants agreed with health professionals about which interventions would be useful for problems described in a vignette. Helpful treatments were based on the consensus of national samples of Australian clinical psychologists, psychiatrists, and GPs [24, 25]. For example, scores for depression ranged from 0 to 6 based on professional consensus that six interventions are helpful (GPs, psychiatrists, clinical psychologists, antidepressants, counselling and cognitive-behaviour therapy).

#### Stigmatising attitudes

Stigmatising attitudes towards people with mental health problems were primarily assessed with Social Distance Scales based on Link et al[26] and Personal Stigma Scales based on Griffiths et al [27]. Other validated stigma scales included the Attitudes to Mental Illness Scale,[28] Opening Minds Scale for Healthcare Providers[29] and the Personal Attributes scale [30].

#### Confidence and intentions

Confidence and intentions to provide MHFA were extracted as proxy measures of behaviour change. Perceived confidence was usually assessed with a single question in relation to helping a person with a mental health problem. This has been shown to be predictive of high quality support provided up to six months later [31]. Intentions to provide mental health first aid were assessed in response to a person with a mental health problem described in a vignette. Studies either used a list of actions consistent with the ALGEE action plan, or invited open-ended responses which were scored for congruence with the ALGEE action plan as described by Yap et al [32]. These scores have high inter-rater reliability [32, 33] and have been shown to predict the quality of support provided up to 2 years later [32].

#### Provision of mental health first aid

This was assessed in two ways in included studies: the amount of help or mental health first aid provided to someone with a mental health problem, and the quality of first aid provided. Studies measured amount of help either on frequency scales (e.g. “never” to “many times”) or whether help had been provided at all (yes/no). Quality was assessed by summing the number of helpful actions participants reported taking to support a person with a mental health problem, which were consistent with MHFA training.

Secondary outcomes collected were:

1. Mental health of MHFA trainees, assessed using a validated measure of depression, anxiety, or psychological distress/internalising symptoms.
2. Mental health of *recipients* of mental health first aid (via MHFA trainees), assessed using a validated measure of depression, anxiety, or psychological distress/internalising symptoms.

Where outcomes were reported as both continuous (e.g. means and standard deviations) and dichotomous (e.g. percentage above a cut-off), we extracted the continuous data. Where studies reported both endpoint data and change over time data (e.g. group by time interactions) we extracted the change over time data due to the potential for baseline imbalances. Where intentions, confidence or behavioural outcomes towards multiple people were reported (e.g. students AND colleagues), we extracted data for the person who was the clearly intended recipient of MHFA (i.e. school students for Youth MHFA participants).

Risk of bias was assessed using the revised Cochrane Risk of Bias tool for randomised trials (RoB 2.0) [34]. This has five domains and assesses bias due to the randomization process, deviations from intended interventions, missing outcome data, measurement of the outcome, and selection of the reported result. An additional domain is included for cluster RCTs which assesses bias arising from the timing of identification and recruitment of individual participants in relation to the timing of cluster randomization. On each domain, risk of bias was judged as low risk, some concerns, or high risk. Studies are rated as high risk of bias overall if at least one domain is judged as high risk of bias. Risk of bias judgements were made independently by two review authors, with discrepancies resolved by consensus. The quality of evidence for all outcomes was judged using the Grading of Recommendations Assessment, Development and Evaluation (GRADE) system [35, 36]. Five factors may lead to rating down the quality of evidence (risk of bias, inconsistency, indirectness, imprecision, and publication bias) and three factors may lead to rating up (large effect size, dose–response relationship, and consideration of all plausible confounding variables). The quality of evidence for each outcome across studies falls into one of four categories: high, medium, low, or very low.

### Data synthesis

Outcomes were analysed with Comprehensive Meta-Analysis (CMA) V2 software using a random-effects model and reported as standardized mean differences. Separate meta-analyses were conducted to examine effects at 3 different pre-specified time-points (post-intervention, up to 6 months post-intervention, greater than 6 months post-intervention). Multiple validated outcomes of the same construct (e.g. attitudes assessed with personal stigma scales and social distance scales) within a study were combined into one effect size using CMA, unless these data were already pooled and reported by trial authors. Where studies reported an effect size for more than one independent subgroup in their sample (e.g. depression vignette versus schizophrenia vignette), we included each subgroup as a separate ‘study’. When interpreting mean effect sizes, we followed Cohen’s guidelines whereby *d* of 0.2 = small, 0.5 = medium, and 0.8 = large [37].

To explore the robustness of the results we performed several pre-specified sensitivity analyses. We tested the impact on pooled estimates of removing studies conducted by the developers of MHFA, studies judged to have high risk of bias, and outlier studies (studies whose 95% CI did not overlap with the 95% CI of the pooled effect size).

Statistical heterogeneity for each pooled outcome was examined with the I^2^ statistic, which expresses the amount of heterogeneity in effect sizes in percentages. A percentage of 25% indicates low heterogeneity, 50% moderate and 75% high heterogeneity [38]. The 95% CI around I^2^ was calculated using the ‘heterogi’ module in STATA. Where I^2^ was not zero and the confidence interval included high levels of heterogeneity, further subgroup and meta-regression analyses were conducted to explore possible causes of heterogeneity. Subgroup analyses used a mixed-effects model (a random-effects model within subgroups and a fixed-effect model across subgroups) and explored whether mean effect sizes differed for face-to-face versus online delivery, standard adult course versus youth MHFA, and type of comparison condition. When there were at least 3 studies per analysis, meta-regression explored the effect of the percentage of female participants and program length.

Small study effects (e.g. publication bias) were assessed when there were 10 or more studies in the meta-analysis by visually examining the funnel plot, supplemented by Egger’s test of funnel plot asymmetry [39, 40]. Duval and Tweedie’s trim and fill procedure was used to estimate the effect size after imputing potentially missing studies [41].

## Results

Searches generated 611 records of which 34 were assessed for full-text eligibility (see Fig 1). Sixteen studies were excluded at the full-text screening stage: 11 were not an MHFA program, 4 were not an RCT or controlled trial, and 1 was a duplicate publication. A final 18 studies were included in the review, with a total of 5936 participants [13–22, 42–49]. Only 6 of these were included in the prior review of MHFA [13, 17–19, 45, 48], with 12 additional controlled trials identified. Study characteristics are detailed in Table 1.

**Table 1:** Characteristics of included studies.

Study	Program	Program length (hours)	N sessions	Mode of delivery	Study design	Comparison condition	Study population	% female	Mean age (SD), range	Education level	N (INT, CON)^b^	Follow-up points	Country	Involvement of MHFA founder
**Burns 2017**	MHFA for nursing students	13	2	face-to-face	RCT	Waitlist	First year undergraduate nursing students	83.6	N/R (N/R), 18–41+	All first year undergraduates	92, 89	post, 2 months	Australia	N
**Davies 2016**	MHFA for adults	6–8	N/A	online/CD-ROM	RCT	No intervention	Medical students	65.5	19.9 (3.2), 18–39	first, second or third year undergraduates	27, 28	post	United Kingdom	N
**Jensen 2016**	MHFA for adults, translated into Danish	12	2	face-to-face	RCT	Waitlist	Danish employees	83.8^a^	43.0^a^ (12.1), N/R	88.0%^a^ with a university degree	290, 276	6 months	Denmark	N
**Jorm 2004**	MHFA for adults	9	3	face-to-face	c-RCT	Waitlist	Members of the public in rural Australia	81.9^a^	47.5^a^ (N/R), N/R	22.0%^a^ with a university degree	416, 337	4 months	Australia	Y
**Jorm 2010a**	MHFA for adults	N/A	N/A	online/ CD-ROM	RCT	Other mental health education intervention: Sent the MHFA manual and received 4 weekly reminder emails to read it.	Members of the public	81.0	40 (12), N/R	56.0% with a university degree	90, 88	post, 6 months	Australia	Y
**Jorm 2010b**	modified version of Youth MHFA	14	2	face-to-face	c-RCT	Waitlist	High school teachers	65.1	N/R (N/R), N/R	N/R	283, 140	post, 6 months	Australia	Y
**Jorm unpublished**	Youth MHFA	14	2–4	face-to-face	RCT	Generic health education intervention: 15-hour Australian Red Cross Apply First Aid course delivered in groups over 2 days or 4 x 3.5hr sessions	Parents of teenagers aged 12–15 years	88.2	45.2 (5.6), 31–67	53.8% with a university degree	202, 183	1 year	Australia	Y
**Kitchener 2004**	MHFA for adults	9	3	face-to-face	RCT	Waitlist	Employees of government departments	78.1	N/R (N/R), 18–60+	60.6% with a university degree	146, 155	5 months	Australia	Y
**Lipson 2014**	MHFA for adults	12	N/R	face-to-face	c-RCT	No intervention	University students	57.5^a^	20.4^a^ (1.3), N/R	Second year or higher university students	535, 507	2–3 months	United States	N
**Massey 2014**	MHFA for adults	12	N/R	face-to-face	CT	No intervention	Student support staff at a university	N/R	N/R (N/R), N/R	N/R	29, 55	6 months	Canada	N
**Moffitt 2014**	MHFA for adults	N/R	2	face-to-face	RCT	Other mental health education intervention: Looking after Wellbeing at Work (LWW) 2 day training course. Locally-developed specifically for the fire service with emphasis on mental health in the workplace.	Fire Service managers	N/R	N/R (N/R), N/R	N/R	41, 31^c^	post	United Kingdom	N
**Mohatt 2017**	Military MHFA, adapted for the military population	8	1	face-to-face	c-RCT	No intervention	Members of the Army National Guard and community first responders	N/R	N/R	N/R	69, 107	8 months	USA	N
**Moll unpublished**	MHFA for adults	12	2	face-to-face	RCT	Other mental health education intervention: Beyond Silence, a new program customised to the healthcare workforce. 12 hours of group-based and online education, co-led by peer educators with experience of mental health problems.	Healthcare employees	88.5	N/R (N/R), 18–69	N/R	108, 108	post, 3 months	Canada	N
**O'Reilly 2011**	MHFA for adults	12	N/R	face-to-face	CT	No intervention	Pharmacy university students	64.0	21 (1.99), 19–35	Third-year undergraduate students	60, 212	6 weeks	Australia	N
**Reavley unpublished**	MHFA for adults, tailored to public service	6	N/A	online/ CD-ROM	RCT	Generic health education intervention: 4 hour elearning version of Apply First Aid. Both groups received weekly emails for 6 weeks	Employees of Victorian Public Service	75.5	41 (10.9), 18–68	66.7% with a university degree	199, 210	post	Australia	Y
**Rose 2017**	Youth MHFA	8	2	face-to-face	CT	No intervention	Social work students	94.5	27.3^a^ (6.0), N/R	All master's level social work students	39, 34	5 months	USA	N
**Svensson 2014**	MHFA for adults, translated into Swedish	12	2	face-to-face	RCT	Waitlist	Swedish employees	77.1^a^	45.6^a^ (10.5), N/R	75.1%^a^ with a university degree	199, 207	6 months	Sweden	N
**Wong 2017**	MHFA for adults, translated into Chinese	12	N/R	face-to-face	CT	Other: Educational programs on stress management and physical health enhancement, equivalent to 12 hours	Members of the public	68.6^a^	N/R (N/R), 18–65	64.6%^a^ with a university degree	161, 183	post, 6 months	Hong Kong, China	N

Note. CT = controlled trial, cRCT = cluster randomised controlled trial, RCT = randomised controlled trial, N/A = not applicable, N/R = not reported, Y = Yes, N = No

^a^Calculated by pooling across conditions

^b^Number randomised

Number analysed rather than randomised

There were 4 cluster-RCTs, 10 RCTs, and 4 controlled trials. Studies used a variety of comparison conditions, with 6 using a waitlist condition, 6 using another health or mental health education program, and 6 using a no intervention design. Outcomes were assessed post-intervention in 8 studies, up to 6 months follow-up in 13 studies (14 comparisons), and only 2 studies examined effects at more than 6 months post-intervention (up to 1 year follow-up). Three studies were unpublished or ongoing [16, 20, 21].

Most studies evaluated the adult course or a variant (k = 15) rather than youth MHFA (k = 3) and delivered the course in a face-to-face format (k = 15) rather than online or CD-ROM (k = 3). The program was evaluated in several settings, including workplaces (k = 7), with university or heath care students (k = 5), members of the public (k = 3), teachers (k = 1), parents (k = 1), and the military (k = 1). Study participants tended to be female (median = 78.1%, range 57.5% - 94.5%). The majority of studies were conducted in Australia (k = 8), with 5 conducted in North America, 4 in Europe/UK, and 1 in Hong Kong. Most studies did not involve the founders of MHFA in their evaluation (k = 12).

### Risk of bias in included studies

**Fig 2:**
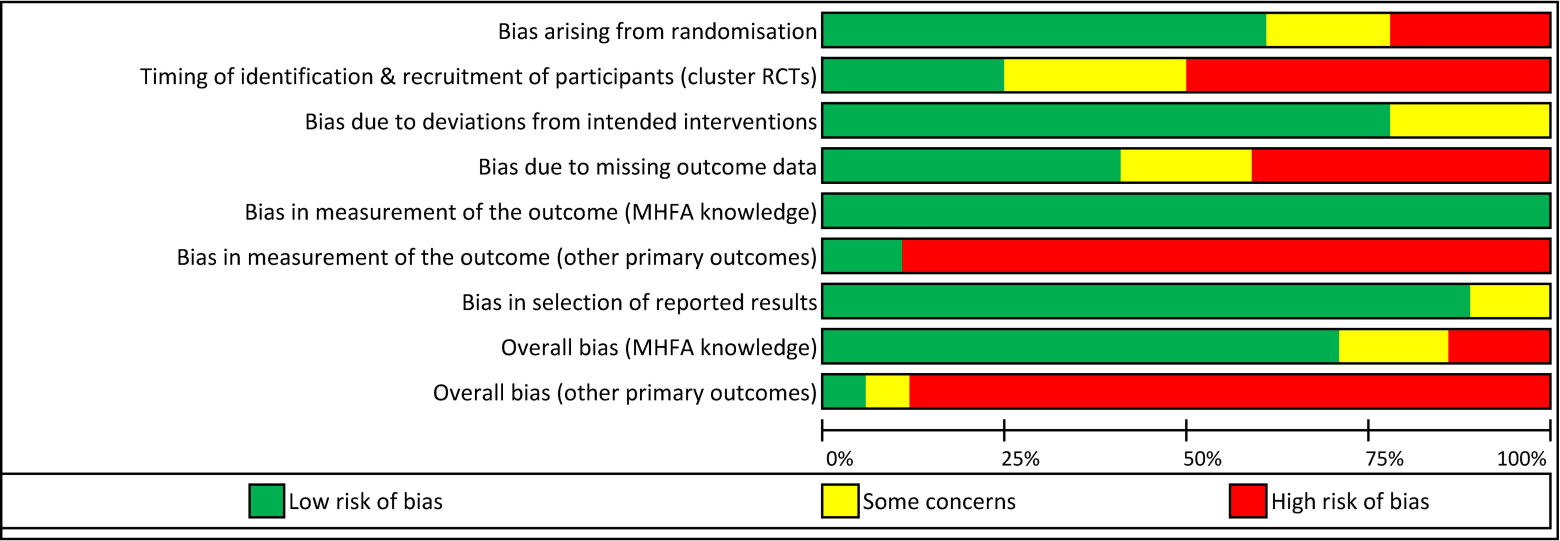
Summary of risk of bias judgements presented as percentages across all included studies.

**Fig 3:**
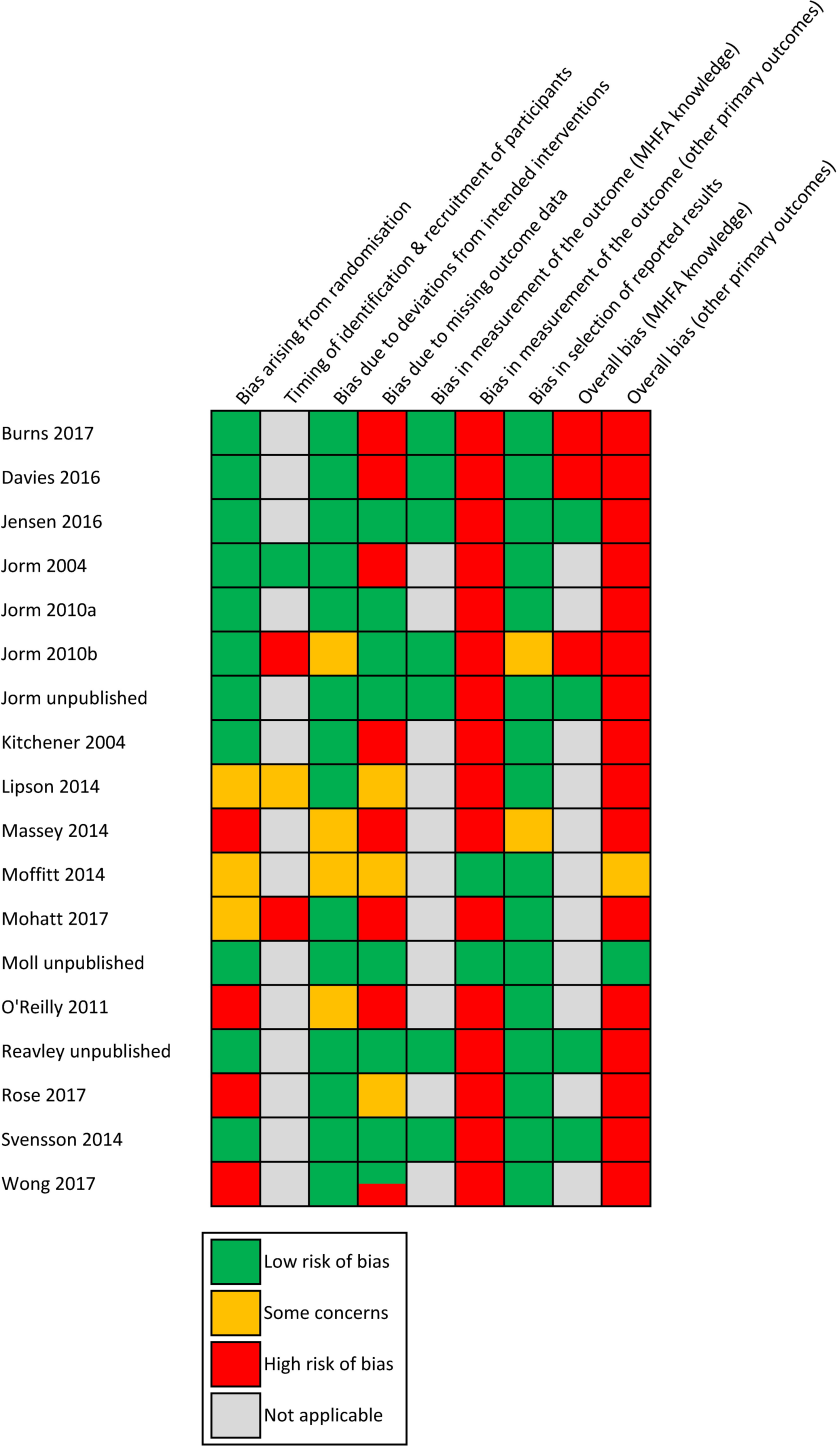
Summary of risk of bias judgements for each study.

Risk of bias was rated per outcome according to Cochrane guidelines[34] and is summarised in Figs 2 and 3. The majority of studies were rated as low risk of bias arising from randomisation; having adequate random sequence generation and allocation concealment and few baseline imbalances. There was generally low risk of bias due to deviations from intended interventions, as most studies reported delivery of MHFA consistent with usual practice and analysed participants in the group they were randomly allocated. Most studies were judged as low risk of bias in the selection of reported results, with 10 studies prospectively registering trial protocols. However, less than half the studies were judged as low risk of bias due to missing data, with 7 studies reporting different proportions of missing data between groups, and inadequate statistical methods for handling missing data, such as analysing complete cases or using last outcome carried forward imputation [34]. As all outcome measures were self-reported, most studies had a high risk of bias in measurement of the outcome. This is because participants were aware of their allocation and may have had expectations of benefit from the MHFA intervention, which may have influenced outcome measurement. Studies that included a mental health education condition as their comparison were rated as low risk of bias, as expectations of benefit would be similar across groups. The assessment of MHFA knowledge, which was an ‘objective’ test of knowledge via true or false answers, was also less likely to be influenced by knowledge of intervention received, hence this outcome was rated as low of bias for all 7 studies that included it. For all other primary outcomes, only one study was rated as low risk of bias overall.

### Effects of MHFA

#### Knowledge

As shown in Table 2, accurate identification of mental health problems showed small non-significant improvements at post-intervention (*d* = 0.22), with low-to-moderate heterogeneity, increasing to moderate improvements by 6-month follow-up (*d* = 0.52) with low heterogeneity. Effect sizes were similar with the removal of 3 studies involving the developers of MHFA at post-intervention, *d* = 0.39 (95% CI: -0.04, 0.81) and 4 studies at follow-up, *d* = 0.43 (95% CI: 0.28, 0.58). There was no evidence of publication bias in the 11 studies at follow-up < = 6 months (Egger’s test, two-tailed p = .271).

**Table 2:** Results of meta-analyses of effects of mental health first aid training on outcomes.

	N comparisons (k)	Cohen's d [95% CI]	*p*	I^2^% [95% CI]	Quality of the evidence (GRADE)
**MHFA knowledge**					
Post	3	0.72 [0.59, 0.86]	**<.001**	0 [0, 90]	⊕⊕⊕⊕ High
< = 6-month follow-up	5	0.54 [0.43, 0.64]	**<.001**	0 [0, 79]	⊕⊕⊕⊕ High
>6-month follow-up	1	0.31 [0.09, 0.53]	**.006**		⊕⊕⊕⊝ Moderate^b^
**Recognition of mental health problem**					
Post	7	0.22 [-0.17, 0.61]	.276	40 [0, 75]	⊕⊕⊝⊝ Low^a^^,^^b^
< = 6-month follow-up	11	0.52 [0.41, 0.64]	**<.001**	11 [0, 51]	⊕⊕⊕⊝ Moderate^a^
>6-month follow-up	1	0.22 [-0.36, 0.80]	.450		⊕⊕⊝⊝ Low^a^^,^^b^
**Beliefs about effective treatments**					
Post	4	0.45 [0.11, 0.79]	**.010**	85 [63, 94]	⊕⊕⊕⊝ Moderate^a^
< = 6-month follow-up	11	0.19 [0.07, 0.32]	**.002**	59 [20, 79]	⊕⊕⊕⊝ Moderate^a^
**Stigma**					
Post	8	0.14 [0.03, 0.25]	**.010**	0 [0, 68]	⊕⊕⊕⊝ Moderate^a^
< = 6-month follow-up	14	0.14 [0.05, 0.23]	**.003**	48 [4, 72]	⊕⊕⊕⊝ Moderate^a^
>6-month follow-up	1	0.08 [-0.14, 0.31]	.473		⊕⊕⊝⊝ Low^a^^,^^b^
**MHFA confidence**					
Post	7	0.58 [0.29, 0.87]	**<.001**	79 [57, 90]	⊕⊕⊕⊝ Moderate^a^
< = 6-month follow-up	12	0.46 [0.31, 0.62]	**<.001**	77 [60, 87]	⊕⊕⊝⊝ Low^a^^,^^c^
>6-month follow-up	2	0.21 [-0.09, 0.51]	.175	28^#^	⊕⊕⊝⊝ Low^a^^,^^b^
**MHFA intentions**					
Post	4	0.75 [0.60, 0.91]	**<.001**	0 [0, 85]	⊕⊕⊕⊝ Moderate^a^
< = 6-month follow-up	3	0.55 [-0.08, 1.18]	.085	91 [76, 96]	⊕⊕⊝⊝ Low^a^^,^^b^
>6-month follow-up	2	0.26 [-0.12, 0.64]	.182	48^#^	⊕⊕⊝⊝ Low^a^^,^^b^
**Amount of MHFA provided**					
Post	3	-0.06 [-0.32, 0.20]	.650	0 [0, 90]	⊕⊕⊝⊝ Low^a^^,^^b^
< = 6-month follow-up	9	0.23 [0.08, 0.38]	**.002**	63 [23, 82]	⊕⊕⊕⊝ Moderate^a^
>6-month follow-up	0				
**Quality of MHFA provided**					
Post	2	0.73 [-0.97, 2.43]	.399	94^#^	⊕⊕⊝⊝ Low^a^^,^^b^
< = 6-month follow-up	2	-0.01 [-0.22, 0.19]	.950	76^#^	⊕⊕⊕⊝ Moderate^b^
>6-month follow-up	1	0.25 [-0.17, 0.24]	.242		⊕⊕⊝⊝ Low^a^^,^^b^
**MHFA trainee mental health**					
Post	3	-0.04 [-0.39, 0.32]	.835	4 [0, 90]	⊕⊕⊝⊝ Low^a^^,^^b^
< = 6-month follow-up	4	0.16 [-0.03, 0.35]	.093	0 [0, 85]	⊕⊕⊝⊝ Low^a^^,^^b^
>6-month follow-up	1	0.02 [-0.20, 0.24]	.882		⊕⊕⊝⊝ Low^a^^,^^b^
**MHFA recipient mental health**					
Post	0				
< = 6-month follow-up	2	0.14 [-0.05, 0.33]	.144	0^#^	⊕⊕⊝⊝ Low^a^^,^^b^
>6-month follow-up	1	-0.09 [-0.32, 0.14]	.458		⊕⊕⊕⊝ Moderate^b^

# too few studies to calculate confidence interval.

GRADE Working Group grades of evidence:

**High quality** ⊕⊕⊕⊕: Further research is very unlikely to change our confidence in the estimate of effect.

**Moderate quality** ⊕⊕⊕⊝: Further research is likely to have an important impact on our confidence in the estimate of effect and may change the estimate.

**Low quality:** ⊕⊕⊝⊝Further research is very likely to have an important impact on our confidence in the estimate of effect and is likely to change the estimate.

**Very low quality:** ⊕⊝⊝⊝We are very uncertain about the estimate.

a Downgraded due to risk of bias.

b Downgraded due to imprecision.

c Downgraded due to publication bias.

Beliefs about effective treatments for mental health problems significantly improved at post-intervention (*d* = 0.45) and at up to 6-month follow-up (*d* = 0.19) with moderate-to-high heterogeneity in effect sizes. Effects were robust in sensitivity analyses exploring researcher allegiance post-intervention, d = 0.91 (95% CI: 0.69, 1.13, k = 1) and follow-up, d = 0.17 (95% CI: 0.00, 0.35, k = 7). Removal of one outlier study[22] at follow-up slightly reduced the effect size, *d* = 0.16 (95% CI: 0.07, 0.24). There was no evidence of publication bias in the 11 studies at follow-up < = 6 months (Egger’s test, two-tailed *p* = .450).

There was a moderate-to-large significant improvement in MHFA knowledge at post-intervention (*d* = 0.72), which was smaller at 6-month follow-up (*d* = 0.54) and 12 month follow-up (*d* = 0.31), with no heterogeneity. Removing studies involving the developers of MHFA increased the effect size at post-intervention to *d* = 0.91 (95% CI: 0.55, 1.26, k = 1) and *d* = 0.53 (95% CI: 0.41, 0.65, k = 4) at 6-month follow-up. Mean effect sizes were the same when removing studies at high risk of bias, d = 0.72 (95% CI: 0.44, 1.00, k = 1) at post-intervention and d = 0.54 (95% CI: 0.42, 0.67, k = 3) at follow-up less than 6 months.

#### Attitudes

There were small significant effects on stigmatizing attitudes at post-intervention (*d* = 0.14) and up to 6-month follow-up (*d* = 0.14), reducing to very small non-significant effects at 12-month follow-up (*d* = 0.08). Heterogeneity was low at post-intervention and moderate at 6-month follow-up. Effects were weaker when removing studies involving the founders of MHFA at post-intervention, *d* = 0.09 (95% CI: -0.05, 0.23, k = 5) and 6-month follow-up, *d* = 0.11 (95% CI: 0.00, 0.21, k = 10), but confidence interval limits overlapped. Similarly, removing one outlier study[48] at follow-up reduced the size of the pooled effect size to *d* = 0.09 (95% CI: 0.03, 0.16). Visual inspection of the funnel plot suggested some asymmetry in effects at 6-month follow-up (Egger’s test, two-tailed *p* = .113). The Duval & Tweedie trim-and-fill approach suggested that four studies were potentially missing and, if imputed, the mean effect size would drop to *d* = 0.08 and would no longer be significant (95% CI: -0.02, 0.18).

Supplementary analyses were conducted to explore whether effects varied depending on the type of stigma, as most studies included measures of both social distance and personal stigma. The mean effect size at post-intervention was *d* = 0.35 (95% CI: -0.08, 0.78) for social distance (k = 4) and *d* = 0.12 (95% CI: -0.06, 0.29) for personal stigma (k = 8). Similarly, at up to 6 months follow-up, effect sizes tended to be larger for social distance, *d* = 0.23 (95% CI: 0.11, 0.36, k = 11) than for personal stigma, *d* = 0.06 (95% CI: -0.02, 0.15, k = 13).

#### Confidence and intentions

There were moderate significant improvements in confidence in helping someone with a mental health problem at post-intervention (*d* = 0.58) and follow-up by 6 months (*d* = 0.46), with high levels of heterogeneity. Effects beyond 6 months were small in size (*d* = 0.21). There were two outlier studies at 6-month follow-up [44, 45]; removing these only slightly reduced the pooled effect size to *d* = 0.43 (95% CI: 0.30, 0.57). Results were robust to researcher allegiance, with very similar effects at post (*d* = 0.59, 95% CI: 0.18, 1.00, k = 4) and < = 6-month follow-up (*d* = 0.48, 95% CI: 0.28, 0.68, k = 8). However, inspection of the funnel plot for the 12 studies at 6-month follow-up suggested evidence of asymmetry (Egger’s test, two-tailed *p* = .008). The Duval & Tweedie trim-and-fill approach suggested that four studies were potentially missing and, if imputed, the mean effect size would drop to *d* = 0.32 (95% CI: 0.15, 0.48).

Effects on intentions to provide mental health first aid were moderate-to-large at post-intervention (*d* = 0.75 with no heterogeneity) and by 6-month follow-up (*d* = 0.55 with high heterogeneity), with smaller effects observed at longer follow-up (*d* = 0.26 with moderate heterogeneity). Effects were somewhat larger when removing studies with MHFA developer involvement at post (*d* = 0.93, 95% CI: 0.63, 1.23, k = 2), up to 6-month follow-up (*d* = 1.04, 95% CI: 0.58, 1.50, k = 1), and beyond 6-months (*d* = 0.55, 95% CI: -0.00, 1.10, k = 1).

#### Behaviour

There were no improvements in the amount of help provided to a person with a mental health problem at post (*d* = -0.06, no heterogeneity), but small improvements were evident at less than 6-month follow-up (*d* = 0.23, moderate heterogeneity). Effects remained similar when removing studies with MHFA founder involvement, post *d* = -0.14 (95% CI: -0.44, 0.15) and follow-up *d* = 0.27 (95% CI: 0.08, 0.46).

The quality of mental health first aid provided to a person with a mental health problem showed a moderate, non-significant improvement at post-intervention (*d* = 0.73, high heterogeneity), but no effects were evident at less than 6-month follow-up (*d* = -0.01, high heterogeneity). One study found small, non-significant improvements at 12-month follow-up (*d* = 0.25).

#### Mental health

Improvement in the mental health of MHFA trainees was not evident at post-intervention (*d* = -0.04) or by 6-month follow-up (*d* = 0.16), with no evidence of heterogeneity at either time point. One study showed no effects at 12-month follow-up (*d* = 0.02). At follow-up less than 6 months, effects were slightly lower when removing three studies that involved the founders of MHFA, *d* = 0.11 (95% CI: -0.40, 0.62).

Three studies evaluated the effect of MHFA training on recipients of MHFA (via MHFA trainees), and found a small non-significant effect by 6-month follow-up (*d* = 0.14, no heterogeneity), and a non-significant negative effect at 12 month follow-up (*d* = -0.09). At follow-up less than 6 months, removing the one study that involved the founders of MHFA and was at high risk of bias had little impact on the effect size, *d* = 0.17 (95% CI: -0.05, 0.39).

### Possible moderators

Where I^2^ was not zero and the confidence interval included high levels of heterogeneity, we investigated possible reasons for variations in effect sizes according to pre-specified study characteristics: type of comparison condition, program delivery format (face-to-face versus online), and program type (adult versus youth). Full results are presented in S1 Table. Subgroup analyses revealed a difference in mean effect sizes between comparison conditions for beliefs about effective treatments at post-intervention (*p*< .001) and follow-up less than 6 months (p = .005), MHFA confidence at post-intervention (p < .001), and amount of MHFA provided at less than 6-month follow-up (p < .001). Studies that included another mental health education intervention as the comparison condition consistently had smaller mean effect sizes than those using a waitlist, no intervention, health education intervention or other form of comparison condition.

For outcomes with at least 3 studies we investigated whether gender and program length were possible sources of heterogeneity in separate meta-regression analyses. There were no consistent effects across outcomes (see S2 Table). Longer programs were associated with lower scores on beliefs about effective treatments at follow-ups less than 6 months (*b* = -0.06, 95% CI: -0.12, 0.00) and studies with a higher proportion of females were associated with lower amounts of MHFA provided to a person with a mental health problem at follow-ups less than 6 months (*b* = -0.01, 95% CI: -0.02, 0.00). Given the lack of consistency in effects, potential for Type II errors from the number of analyses conducted, and the observational nature of meta-regression, these findings should be interpreted with caution.

## Discussion

This review identified 18 controlled trials evaluating the effectiveness of MHFA training in a variety of settings. Across primary outcomes, there were generally small to moderate improvements at post-training and up to 6 months later. Effects at up to 12-month follow-up were less clear and require further replication. For knowledge outcomes, there is strong evidence that the training improves knowledge about mental health problems, with effects persisting up to a year after training. It also leads to moderate improvements in beliefs about appropriate treatments. Accurate identification of a person with a mental health problem improved up to 6 months later, but effects were smaller immediately after training and it is unclear why this was the case. MHFA training led to approximately small reductions in stigmatising attitudes, with post-hoc analyses suggesting greater reductions in social distance attitudes than other measures of stigma. Perceived confidence in helping a person with a mental health problem was largest post-training, but moderate effects persisted up to 6 months later. At post-training there were moderate-to-large improvements in intentions to provide first aid to a person with a mental health problem, which reduced at follow-up. Perhaps unsurprisingly, there were no changes in the amount of MHFA provided to others at post-training, given the short duration of the course and lack of opportunity to assist others. However, small effects were evident by 6-month follow-up, with heterogeneity explained by the lack of effect in studies comparing MHFA with another mental health education intervention. The impact of MHFA training on the quality of behaviours offered to a person with a mental health problem is less clear, as the pooled effect size confidence intervals failed to rule out no improvement or less improvement compared with controls. Effects on secondary outcomes–the mental health of trainees and MHFA recipients–were less convincing. There were potentially very small effects at up to 6-month follow-up, but estimates were imprecise and may change with further research.

Overall, the effects of MHFA training were robust to researcher allegiance, as effects were similar when excluding studies conducted by the developers of MHFA. This suggests that outcomes will be similar if evaluated by different research groups in different countries, and supports the program’s spread worldwide. Several outcomes showed significant variation in effect sizes across studies, which was often explained by the choice of comparison condition. Studies that compared MHFA with a different mental health education intervention generally showed no significant difference in effects. Other subgroup analyses investigating the effect of delivery format and program type were inconclusive due to a lack of power. With the exception of MHFA knowledge, the quality of the evidence was generally moderate or low, typically due to lack of blinding or potential bias from missing outcome data. It is difficult to achieve blinding for this type of intervention, as it requires a plausible control condition. The combination of self-reported outcomes with participants aware of whether they were in the intervention or control meant that most studies suffered potential bias in measurement of outcomes. This limitation could be overcome with an objective measure of MHFA behaviours, such as a simulated encounter with a person with a mental health problem, as has been used to evaluate therapist competence [50]. Nevertheless, positive changes to actual helping behaviour from MHFA training is supported by qualitative research that gathered stories from MHFA trainees approximately 20 months after training [51].

Results were generally consistent with those reported in an earlier review of MHFA that included fewer controlled trials [9] and 6 overlapping studies. Estimates are not directly comparable as the earlier review pooled together post-training and follow-up effects. For knowledge outcomes, Hadlaczky and colleagues found a small-to-moderate improvement (g = 0.38, 6 trials) in recognition of mental health problems and beliefs about effective treatments. Improvements in stigma (social distance) were somewhat higher than those reported here, but are comparable with our supplementary analyses restricted to social distance outcomes specifically. Similarly, effects on helping behaviour (g = 0.24, 5 trials) were very similar to the amount of MHFA provided at follow-up from 9 trials in our analyses. Consistent with prior literature [52], our analyses found greater effects for intentions to provide help than actual help provided. Nevertheless, evidence indicates that intentions to provide MHFA do predict actual behaviour provided to a person with a mental health problem later on (r = 0.27) [31].

This review’s strengths were its rigorous methodology and comprehensive inclusion of relevant studies, including 3 unpublished or ongoing studies. We extracted the full range of outcomes from MHFA training and conducted a detailed evaluation of risk of bias of each study outcome. Limitations included the small number of studies in some analyses, which affected the precision of pooled estimates and limited the power to investigate heterogeneity in subgroup and meta-regression analyses.

This review has identified some evidence gaps that could be further investigated. Only two studies have thus far examined MHFA training effects beyond 6 months [16, 47], hence the persistence of effects in the longer-term is unclear. Few studies have examined the quality of mental health first aid behaviours, that is, how well trainees actually provided support or assistance to a person with a mental health problem. This is arguably the key outcome of interest from the training, but is more difficult to assess than other outcomes. It requires participants to have had contact with someone with a mental health problem, and to describe in sufficient detail what actions they took. Assessing this over a longer follow-up period would mean there would be more opportunities to provide help, and greater statistical power to show an impact from the training. As the ultimate aim of MHFA training is to improve mental health outcomes, collecting data on the recipients of mental health first aid behaviours is an important goal. Although few studies in this review achieved this, it is acknowledged that collecting this data is challenging because recipients of MHFA support are not usually study participants.

Although effects were generally small to moderate, MHFA training could potentially have a large public health impact. Evidence suggests that many people are not well informed about how to recognise mental health problems in others, how to respond to them, and what services and effective treatments are available [53]. Stigmatising attitudes about mental health problems may also impact on treatment seeking and adherence and increase social exclusion [54, 55]. MHFA training offers more than just facts about mental health problems and myth-busting; it provides concrete steps and advice on how to approach and support a person with a mental health problem. This is important as avoidance and lack of understanding are key experiences of discrimination reported by those with common mental disorders [56]. Given low rates of treatment-seeking [57], and evidence that people are more likely to seek help if someone close to them suggests it [58, 59], the support that people receive from those in their social networks is an important factor in improving mental health outcomes.

In conclusion, this review supports the effectiveness of MHFA training in improving mental health literacy and appropriate support for those with mental health problems. This public health intervention is a noteworthy contribution to improving the lives of people with mental health problems.

## Supporting information

S1 Table Results of sub-group analyses investigating causes of heterogeneity in effect sizes in studies evaluating MHFA. (PDF)

S2 Table Meta-regression analyses of percentage of female participants and length of program as predictors of effect sizes in studies examining the effect of MHFA. (PDF)

S3 Table Systematic search terms. (DOCX)

S4 Table PRISMA checklist. (PDF)
