# Supplementary material for: Systematic review and meta-analysis of Mental Health First Aid training: Effects on knowledge, stigma, and helping behaviour
Source: PLoS One. 2018 May 31;13(5):e0197102. doi: 10.1371/journal.pone.0197102 (PMC5979014; doi:10.1371/journal.pone.0197102)
Supplement: S1 Table — (PDF) [file pone.0197102.s001.pdf]

S1 Table. Results of sub-group analyses investigating causes of heterogeneity in effect sizes in studies evaluating MHFA

					Difference between subgroups		
	Number of Comparisons (k)	Cohen's d [95% CI]	*p*	I^2 % [95% CI]	Q-value	df (Q)	*p* for Q-value
**Recognition of mental health problem - Post**							
Comparison condition					0.93	4	.920
Waitlist	2	0.04 [-0.98, 1.07]	.935	0[#]			
No intervention	2	0.06 [-0.98, 1.09]	.910	71[#]			
Generic health education intervention	1	0.15 [-1.25, 1.55]	.835				
Other mental health education intervention	1	-0.24 [-1.89, 1.42]	.780				
Other	1	0.58 [-0.46, 1.61]	.273				
Delivery format					1.24	1	.265
Face to face	3	0.40 [-0.02, 0.82]	.059	28 [0, 93]			
Online	4	-0.01 [-0.59, 0.58]	.980	19 [0, 88]			
Program type					0.27	1	.602
Adult MHFA	6	0.26 [-0.19, 0.71]	.254	37 [0, 75]			
Youth MHFA	1	-0.02 [-0.96, 0.93]	.972				
**Beliefs about effective treatments - post**							
Comparison condition					20.15	3	**<.001**
Waitlist	1	0.29 [0.07, 0.50]	**.008**				
Generic health education intervention	1	0.39 [0.20, 0.59]	**<.001**				
Other mental health education intervention	1	-0.07 [-0.80, 0.67]	.862				
Other	1	0.91 [0.69, 1.13]	**<.001**				

Delivery format					0.71	1	.401
Face to face	2	0.60 [0.03, 1.17]	**.041**	94[#]			
Online	2	0.23 [-0.42, 0.87]	.487	28[#]			
Program type					0.23	1	.635
Adult MHFA	3	0.50 [0.04, 0.97]	**.035**	87 [61, 95]			
Youth MHFA	1	0.29 [-0.45, 1.03]	.445				
**Beliefs about effective treatments - <= 6-month follow-up**							
Comparison condition					12.85	3	**.005**
Waitlist	7	0.15 [0.05, 0.24]	**.002**	30 [0, 70]			
No intervention	2	0.33 [-0.06, 0.72]	.102	0[#]			
Other mental health education intervention	1	-0.09 [-0.81, 0.62]	.795				
Other	1	0.61 [0.36, 0.86]	**<.001**				
Delivery format					0.56	1	.454
Face to face	10	0.20 [0.07, 0.33]	**.002**	62 [24, 81]			
Online	1	-0.09 [-0.86, 0.67]	.809				
Program type					0.03	1	.857
Adult MHFA	10	0.19 [0.05, 0.33]	**.010**	63 [26, 81]			
Youth MHFA	1	0.23 [-0.17, 0.63]	.264				
**Stigma - <= 6-month follow-up**							
Comparison condition					2.27	3	.518
Waitlist	8	0.15 [0.02, 0.29]	**.027**	0 [0, 68]			
No intervention	3	0.22 [0.01, 0.44]	**.039**	85 [54, 95]			

Other mental health education intervention	2	-0.09 [-0.45, 0.27]	.629	69#			
Other	1	0.09 [-0.26, 0.43]	.624				
Delivery format					0.99	1	.320
Face to face	13	0.13 [0.04, 0.22]	**.005**	50 [5, 74]			
Online	1	0.56 [-0.28, 1.40]	.191				
Program type					1.47	1	.226
Adult MHFA	13	0.13 [0.04, 0.22]	**.005**	48 [2, 73]			
Youth MHFA	1	0.51 [-0.10, 1.12]	.103				
**MHFA confidence - post**							
Comparison condition					27.21	4.00	**<.001**
Waitlist	2	1.11 [0.79, 1.43]	**<.001**	0#			
No intervention	1	0.64 [0.10, 1.19]	**.020**				
Generic health education intervention	1	0.24 [0.04, 0.43]	**.017**				
Other mental health education intervention	2	0.16 [-0.10, 0.43]	.228	0#			
Other	1	0.60 [0.38, 0.81]	**<.001**				
Delivery format					0.72	1	.395
Face to face	4	0.70 [0.30, 1.10]	**.001**	86 [64, 94]			
Online	3	0.42 [-0.08, 0.92]	.098	7 [0, 91]			
Program type					2.82	1	.093
Adult MHFA	6	0.51 [0.22, 0.79]	**<.001**	78 [52, 90]			
Youth MHFA	1	1.36 [0.40, 2.31]	**.005**				

**MHFA confidence - <= 6-month follow-up**							
Comparison condition					2.21	3	.529
Waitlist	6	0.52 [0.26, 0.77]	**<.001**	63 [11, 85]			
No intervention	3	0.63 [0.25, 1.01]	**.001**	92 [80, 97]			
Other mental health education intervention	2	0.16 [-0.35, 0.67]	.541	0[#]			
Other	1	0.40 [-0.17, 0.98]	.172				
Delivery format					0.04	1	.850
Face to face	11	0.47 [0.31, 0.63]	**<.001**	79 [63, 88]			
Online	1	0.38 [-0.54, 1.30]	.417				
Program type					2.59	1	.108
Adult MHFA	10	0.42 [0.27, 0.58]	**<.001**	78 [61, 88]			
Youth MHFA	2	0.88 [0.35, 1.41]	**.001**	0[#]			
**MHFA confidence - >6-month follow-up**							
Comparison condition					1.40	1	.237
no intervention	1	0.48 [-0.07, 1.03]	.088				
generic health education intervention	1	0.12 [-0.10, 0.34]	.285				
Program type					1.40	1	.237
Adult MHFA	1	0.48 [-0.07, 1.03]	.088				
Youth MHFA	1	0.12 [-0.10, 0.34]	.285				
**MHFA intentions - <= 6-month follow-up**							
Program type					0.01	1	.933
Adult MHFA	2	0.58 [-0.30, 1.45]	0.198	95[#]			
Youth MHFA	1	0.51 [-0.83, 1.85]	0.457				

**MHFA intentions - >6-month follow-up**							
Comparison condition					1.93	1	.165
no intervention	1	0.55 [-0.00, 1.10]	.051				
generic health education intervention	1	0.13 [-0.09, 0.35]	.254				
Program type					1.93	1	.165
Adult MHFA	1	0.55 [-0.00, 1.10]	.051				
Youth MHFA	1	0.13 [-0.09, 0.35]	.254				
**Amount of MHFA provided - <= 6-month follow-up**							
Comparison condition					17.44	2	**<.001**
Waitlist	5	0.25 [0.14, 0.35]	**<.001**	0 [0, 79]			
No intervention	2	0.43 [0.32, 0.55]	**<.001**	26[#]			
Other mental health education intervention	2	-0.21 [-0.50, 0.09]	.170	0[#]			
Delivery format					2.01	1	.156
Face to face	8	0.25 [0.11, 0.40]	**.001**	63 [19, 83]			
Online	1	-0.39 [-1.27, 0.49]	.381				
Program type					0.11	1	.738
Adult MHFA	8	0.22 [0.07, 0.38]	**.005**	67 [31, 84]			
Youth MHFA	1	0.34 [-0.31, 0.99]	.307				
**Quality of MHFA provided - post**							
Comparison condition					2.76	1	.096
No intervention	1	1.64 [0.10, 3.18]	**.037**				
Other mental health education intervention	1	-0.10 [-1.45, 1.26]	.889				

Delivery format				2.76	1	.096
Face to face	1	-0.10 [-1.45, 1.26]	.889			
Online	1	1.64 [0.10, 3.18]	**.037**			
**Quality of MHFA provided - <= 6-month follow-up**						
Delivery format				1.21	1	.272
Face to face	1	-0.22 [-0.76, 0.31]	.411			
Online	1	0.20 [-0.34, 0.74]	.465			

too few studies to calculate confidence interval
