# Supplementary material for: Systematic review and meta-analysis of Mental Health First Aid training: Effects on knowledge, stigma, and helping behaviour
Source: PLoS One. 2018 May 31;13(5):e0197102. doi: 10.1371/journal.pone.0197102 (PMC5979014; doi:10.1371/journal.pone.0197102)
Supplement: S2 Table — (PDF) [file pone.0197102.s002.pdf]

S2 Table. Meta-regression analyses of percentage of female participants and length of program as predictors of effect sizes in studies examining the effect of MHFA

	% Gender			Length of program (hours)		
	**k**	**Slope point estimate [95% CI]**	***P***	**k**	**Slope point estimate [95% CI]**	***p***
**Recognition of mental health problem**						
Post	7	-0.01 [-0.08, 0.07]	.857	4	0.00 [-0.17, 0.17]	.983
**Beliefs about effective treatments**						
Post	4	-0.03 [-0.11, 0.05]	.468	3	0.01 [-0.14, 0.16]	.901
<=6 month FU	11	-0.01 [-0.03, 0.01]	.301	10	-0.06 [-0.12, 0.00]	**.048**
**Stigma**						
<= 6 month FU	13	-0.01 [-0.02, 0.00]	.255	13	0.00 [-0.07, 0.07]	.993
**MHFA confidence**						
Post	7	-0.02 [-0.05, 0.02]	.336	5	0.09 [-0.03, 0.22]	.142
<= 6 month FU	11	0.01 [-0.01, 0.02]	.398	11	0.02 [-0.09, 0.13]	.730
**MHFA Intentions**						
<= 6 month FU	3	0.01 [-0.08, 0.10]	.863	3	0.13 [-0.10, 0.36]	.278
**Amount of MHFA provided**						
<= 6 month FU	8	-0.01 [-0.02, 0.00]	**.012**	8	0.03 [-0.07, 0.13]	.586
