# Supplementary material for: Systematic review and meta-analysis of Mental Health First Aid training: Effects on knowledge, stigma, and helping behaviour
Source: PLoS One. 2018 May 31;13(5):e0197102. doi: 10.1371/journal.pone.0197102 (PMC5979014; doi:10.1371/journal.pone.0197102)
Supplement: S3 Table — (DOCX) [file pone.0197102.s003.docx]

S3 Table – Systematic search terms

| **Database** | **Mesh & Keyword Search terms** |
| --- | --- |
| **PubMed**  Search performed 19/4/17; re-ran 12/10/2017 | ("Controlled Clinical Trial"[Publication Type] OR (trial OR evaluation OR effective*)) AND ("Mental Disorders"[Mesh] OR "mental health") AND ("First Aid"[Mesh] OR "first aid" OR "mental health training" OR "MHFA" OR "mental health first aid") AND (("2000/01/01"[PDAT] : "2018/12/31"[PDAT]) AND "humans"[MeSH Terms]) |
| **PsycInfo**  Search performed 19/4/17; re-ran 12/10/2017 | (exp *Mental Disorders/ or exp *Mental Health/ or "mental health".mp.) and ("first aid" or "mental health training" or "MHFA" or "mental health first aid").mp. and (exp Clinical Trials/ or (trial or evaluation or effective*)  limit 4 [SEARCH] to (human and yr="2000 -Current") |
| **EMBASE**  Search performed 19/4/17; re-ran 12/10/2017 | (controlled clinical trial/ or (trial or evaluation or effective*)) AND (exp *mental health/ or exp *mental disease/ or "mental health") AND (exp *first aid/ or "first aid".mp. or "mental health training" or "MHFA" or "mental health first aid")  limit 7 [SEARCH] to (human and yr="2000 -Current") |
| **Cochrane Central Register of Controlled Trials**  Search performed 19/4/17; re-ran 12/10/2017 | mental health first aid OR MHFA |
| **International Clinical Trials Registry**  Search performed 19/4/17; re-ran 12/10/2017 | mental health first aid OR MHFA |
